# Supplementary material for: Different Ultrasound Exposure Times Influence the Physicochemical and Microbial Quality Properties in Probiotic Goat Milk Yogurt
Source: Molecules. 2020 Oct 12;25(20):4638. doi: 10.3390/molecules25204638 (PMC7587201; doi:10.3390/molecules25204638)
Supplement: Supplementary file 1 [file molecules-25-04638-s001.pdf]

Table S1. Pearson´s correlation test between microbial count and physicochemical parameters in goat´s milk yogurts stocked at 4 °C.

Parameter	*S. thermophulis*			*L. bulgaricus*			*L. acidophilus* LA-5		
	**Equation**	**R**	**p**	**Equation**	**R**	**p**	**Equation**	**R**	**p**
pH	-0.056 S_t + 4.87	-0.955	**0.010**	-0.040 L_b + 4.59	-0.964	**0.035**	-0,030 L_a + 4,53	-0.952	**0.011**
Lactose	-0.816 S_t + 58.41	-0.664	0.336	-0.517 L_b + 53.96	-0.588	0.412	-0.405 L_a + 53.33	-0.615	0.385
Glucose	-0.001 S_t + 0.122	-0.056	0.944	-0.003 L_b+ 0.126	-0.166	0.834	-0.002 L_a + 0.123	-0.176	0.823
Galactose	0.032 S_t + 0.784	0.836	**0.022**	0.022 L_b + 0.949	0.797	**0.042**	0.016 L_a + 0.981	0.778	**0.045**
Citric	0.001 S_t+ 0.118	0.078	0.921	0.001 L_b + 0.119	0.156	0.844	0.001 L_a + 0.122	0.116	0.884
Lactic	0.0237 S_t + 1.58	-0.291	0.709	-0.011 L_b + 1.43	-0.191	0.809	-0.010 L_a + 1.42	-0.221	0.779
Formic	0.007 S_t+ 0.354	0.929	**0.020**	0.005 L_b + 0.393	0.883	**0.019**	0.004 L_a + 0.400	0.878	**0.020**
Cadaverin	-0.205 S_t+ 4.86	-0.399	0.601	-0.152 L_b + 3.87	-0.414	0.586	-0.125 L_a + 3.71	-0.453	0.547
Putrescine	-0.05847 S_t + 3.21	-0.484	0.516	-0.051 L_b+ 2.96	-0.588	0.412	-0.037 L_a + 2.89	-0.580	0.420
Spermidine	0.414 S_t - 1.78	0.784	0.216	0.273 L_b + 0.412	0.724	0.276	0.211 L_a + 0.758	0.747	0.253
Spermine	0.064 S_t + 1.25	0.591	0.409	0.052 L_b + 1.54	0.676	0.324	0.038 L_a + 1.62	0.654	0.346
Tyramine	0.594 S_t + 10.19	0.612	0.387	0.436 L_b + 13.11	0.629	0.371	0.308 L_a + 13.82	0.593	0.407
Total BA	1.85 S_t + 11.36	0.940	**0.016**	1.26 L_b + 20.99	0.897	**0.015**	0.943 L_a+ 22.78	0.892	**0.016**
Firmness	-0.445 S_t+ 38.64	-0.344	0.656	-0.334 L_b + 36.49	-0.362	0.638	-0.278 L_a + 36.17	-0.402	0.598
Consistency	-2.30 S_t + 382.6	-0.149	0.851	-1.87 L_b + 372.3	-0.170	0.830	-1.75 L_a + 371.5	-0.212	0.788
Cohesiveness	0.408 S_t - 9.14	0.627	0.372	0.280 L_b - 7.03	0.602	0.398	0.222 L_a - 6.71	0.637	0.362
K	-2.97 S_t + 32.46	-0.567	0.432	-2.26 L_b + 18.32	-0.605	0.395	-1.78 L_a + 15.62	-0.635	0.365
n	0.072 S_t - 0.264	0.532	0.468	0.055 L_b+ 0.073	0.575	0.424	0.044 L_a + 0.139	0.606	0.394

K: consistency index; n: flow behavior index; S_t: *Streptococcus thermophilus*; L_b: *Lactobacillus bulgaricus*; L_a: *Lactobacillus acidophilus* LA-5; R: Pearson´s correlation coeficient; p: probability value. Values in bold indicate significance of model ($p < 0.05$).

Table S2. Optimism-corrected performance estimates through validation by bootstrap approach of significant models for prediction of physicochemical parameters from microbial count in goat´s milk yogurt stored at 4 °C during 28 days.

Specie	Parameter	R^2_{app}	R^2_{boot}	R^2_{orig}	Optimism	R^2_v	R_v
S. thermophilus	pH	0.912	0.792	0.893	-0.100	1.012	-1.006
	Galactose	0.699	0.672	0.697	-0.025	0.724	0.851
	Formic acid	0.864	0.892	0.847	0.045	0.819	0.905
	Total BA	0.884	0.892	0.856	0.036	0.848	0.921
L. bulgaricus	pH	0.930	0.751	0.887	-0.135	1.065	-1.032
	Galactose	0.635	0.646	0.507	0.139	0.496	0.704
	Formic acid	0.779	0.827	0.689	0.139	0.640	0.800
	Total BA	0.804	0.830	0.726	0.104	0.700	0.837
L. acidophilus LA-5	pH	0.905	0.763	0.857	-0.094	0.999	-0.999
	Galactose	0.605	0.592	0.596	-0.003	0.608	0.780
	Formic acid	0.771	0.813	0.743	0.070	0.701	0.837
	Total BA	0.796	0.817	0.785	0.032	0.764	0.874

BA: biogenic amine; R^2_{app}: apparent coefficient of determination; R^2_{boot}: bootstrap coefficient of determination; R^2_{orig}: original coefficient of determination; R^2_v: coefficient of determination of the model after validation. R_v: correlation coefficient of the model after validation
